# Renal Hemorrhage

**Published:** 1886-04

**Authors:** William G. Ring

**Affiliations:** Buffalo, N. Y.


### Renal Hemorrhage.*

By William G. Ring, M. D., Buffalo, N. Y.

On the evening of October 7, 1885, Mr. A. N., of this city, presented himself for treatment, and gave the following statement of his case: He said he was eighteen years old; that, bodily, he had developed very fast within the last year; that he was employed in a stone-yard to assist in moving large stones; that several days previous, after lifting, with help of two other men, a stone weighing about 600 pounds, he had experienced severe pain in the lumbar region, and noticed that his urine had become very red in color, apparently due to blood.

He was then examined physically. On passing the finger along the track of the urethra below, from behind forward, no blood escaped from the meatus, and he said no clear blood had escaped that way between the acts of urination. Next, a flexible catheter was introduced into the urethra as far as possible without entering the bladder, and then it was withdrawn. The passage of the catheter was painless, and it showed no trace of blood after withdrawal. The urethra, as a possible source of the hemorrhage, was then considered to be eliminated from the diagnosis. The catheter was then introduced into the bladder, the small bone tip on the end having been removed so that a bulb syringe could be attached. About four ounces of highly-colored urine were drawn off. The end of the catheter was then connected with a Davidson syringe, and about six ounces of tepid water was injected into the bladder and immediately withdrawn, and about eight ounces more thrown in. The six ounces first injected, on withdrawal, were pale compared to the urine. The latter injection was allowed to remain about three minutes, long enough to become mixed with any blood that might come exclusively from the interior of the bladder, but not so long as to become tinged with blood from ureters or kidneys. The water was allowed to flow from the catheter, and came away scarcely tinged. As no pain had been felt in the bladder, no intermit-

*Read before Buffalo Medical and Surgical Association.

tent flow of urine, or any other symptom that seemed to point to it as the source of hemorrhage, the examination proceeded. No examination was made for calculus. The ureters were excluded from purely circumstantial evidence.

First.—The absence of tubular casts, which would most probably have been present had one or both ureters been the source of hemorrhage and perceptible to the unaided eye.

Second.—The fact that the probable cause, viz., the great effort at lifting, which is generally accompanied by temporary cessation of breathing, the lungs being fully inflated, would tend to lessen the tension on the ureters, thus protecting, instead of harming, them.

A test tube, containing an ounce of the colored urine, held between my eyes and light, presented a very uniform appearance with regard to the distribution of the red coloring matter.

The patient was then questioned in regard to his food, whether he had eaten any beets, red cabbage, madder, etc., which might tinge his urine red, to which he gave a negative reply. A sample of the urine was then boiled. It lost its red color, and a large number of brown flocculi formed, floating in a fluid having the light yellow color of normal urine.

As there was only one probable source of the hemorrhage left, that of the kidneys, one or both, most likely from the cortical portion, the diagnosis of renal hemorrhage was made.

The patient was given one ounce of fluid extract of ergot, with directions to take a teaspoonful three times a day—morning, noon, and night—and to cease working. This he did, and came again two days after, bringing a sample of urine much less colored, but not devoid of blood.

A prescription containing

| | | |
|---|---|---|
| Glycerinæ destilat. | | |
| Aq. Pip. Menth., - - - | aa. | 32 grams. |
| Acidi Tannici, - - - - | | 4 " |
| Morphiæ Sulph., - - - - | | .13 " |

was given, one drachm morning, noon, night, and at bedtime, which proved effectual, and he returned to work which required no lifting after one week devoted to treatment, the hemorrhage cured, the urine being free from blood.

The cause of the hemorrhage was probably slight laceration of a small portion of the cortical substance of one or both kidneys, due to his efforts at lifting. During a strong effort of this kind, the breath, deeply inspired, is almost invariably held. As the downward limit of the kidneys' movability is placed at one-half inch, it seems reasonable to infer that the cortical portion of these organs might be slightly lacerated by the downward pressure of the posterior part of the diaphragm, against which they rest. Gray, writing in reference to these points, says that during a deep inspiration both kidneys are depressed by the diaphragm nearly half an inch, and that the renal cortical substance is of a dark red color, dense in structure, and easily lacerable under mechanical force.

Hæmaturia, being merely a symptom, it is to be considered in connection with those obvious divisions of the urinary tract from which the hemorrhage may arise.

1. Hemorrhage from the urethra.
2. Hemorrhage from the prostate and the neck of the bladder.
3. Hemorrhage from the bladder itself.
4. Hemorrhage from the kidneys.
5. Hemorrhage of a general character from the urinary tract.

